# Evaluating association of vaccine response to low serum zinc and vitamin D levels in children of a birth cohort study in Dhaka

**DOI:** 10.1016/j.vaccine.2020.10.048

**Published:** 2021-01-03

**Authors:** Rina Das, Mohammod Jobayer Chisti, Md. Ahshanul Haque, Md. Ashraful Alam, Subhasish Das, Mustafa Mahfuz, Dinesh Mondal, Tahmeed Ahmed

**Affiliations:** Nutrition and Clinical Services Division, icddr,b, Dhaka, Bangladesh

**Keywords:** MAL-ED, Vaccine titer, Ig A, ELISA, WAMI score, ALRI

## Abstract

**Background:**

Vaccine-preventable infectious diseases are often responsible for childhood morbidity and high rates of mortality. Immune response to the vaccine is associated with multiple factors in early childhood and measured by antibody titers. Among them, micronutrient deficiencies such as vitamin D and zinc deficiencies are the most important in resource-limited settings like Bangladesh.

**Objective:**

We aimed to evaluate the association of vaccine response to low serum zinc and vitamin D levels in children.

**Methods:**

We evaluated vaccine response for measles and poliovirus, tetanus and pertussis toxoid, and Ig A antibody levels to rotavirus by ELISA and serum vitamin D and zinc at 7 and 15 months in the MAL-ED birth cohort of the Bangladesh site. By using population-specific generalized estimating equations (GEE), the association between each explanatory variable and the binary outcome variable was examined longitudinally where the dependent variable was vaccine titers and the independent variables were low serum vitamin D and zinc levels.

**Results:**

The GEE multivariable model identified a positive association between serum zinc level and tetanus vaccine titer (OR: 1.84; 95% CI: 1.07–3.17 and p value = 0.028) after adjusting for age, gender, birth weight, WAMI score, diarrhea, ALRI, exclusive breastfeeding, serum ferritin, serum retinol and undernutrition (stunting, wasting, underweight). No association was found between the rest of the vaccine titers with serum vitamin D and zinc level (p > 0.05).

**Conclusion:**

In the MAL-ED birth cohort, where children were followed for five years, serum zinc level had a positive impact on tetanus vaccine titers.

## Highlights

• MAL-ED Bangladesh birth cohort data used in the analysis.
• Relationship between vaccine titers and micronutrient data explored.
• Positive association found between serum zinc level and tetanus vaccine titer.
• The finding implicates the importance of improving zinc nutrition status of the children.

## 1 Introduction

Many infectious diseases can cause childhood morbidity and high rates of mortality but are easily preventable by vaccination. According to the World Health Organization (WHO), each year we can save the lives of>3 million people worldwide and prevent illness and disability by immunization [1]. Improved nutrition and vaccination are the key factors in reducing childhood mortality and morbidity. Immune response to vaccines in early childhood can be measured by antibody titers and depends on multiple factors [2]. The presence or the lack of certain nutrients and vitamins often influence the immune responses [3], [4]. The National micronutrient survey done in Bangladesh in 2011–2012 showed that zinc deficiency was highly prevalent in Bangladeshi children, to the extent of 44.6% among under-five children living in impoverished communities [5]. The prevalence of vitamin A deficiency (serum retinol < 0.7 mmol/l) among children in Bangladesh is also high at 20.5% despite a successful vitamin A supplementation program [5]. Micronutrient deficiencies such as vitamin D and zinc deficiencies can be one of the important factors associated with reduced vaccine response in resource-poor settings like Bangladesh.

The immune function of a child can be assessed by measuring his/her vaccine titers. Several earlier evidence [6] demonstrate that micronutrient deficiencies may have an impact on vaccine response. To date, only some studies have been conducted to detect the vaccine response in children and the effect of vitamin D and zinc deficiencies on it.

Vitamin D deficiency is a common phenomenon among children [7] and this deficiency has been indicated to adversely affect immune responses. Recent evidence has shown that vitamin D is linked with both innate [8] and acquired [9] immune responses and for that reason, vitamin D might have a role in vaccine immunogenicity [10]. The estimated global prevalence of zinc deficiency was 31%, ranging from 4 to 73% [11]. Zinc plays an important role in different aspects of the immune system, extensive studies and reviews have been conducted on the role of zinc on the immune system [12]. Few studies have illustrated the role of zinc deficiency or supplementation on the effect of different vaccine responses [13] in different age groups, but no study has established the relation between zinc deficiency with tetanus, pertussis, measles, rotavirus, and polio vaccine responses.

The association between serum vitamin D and zinc level with vaccine responsiveness remains theoretical and therefore further studies need to be designed. Early identification of vitamin D and zinc deficiency among children may reduce morbidity and mortality due to vaccine-preventable diseases. Here we aimed to evaluate the association of vaccine response to low serum zinc and vitamin D levels in children.

## 2 Methods

### 2.1 Study site and population

**Fig. 1:**
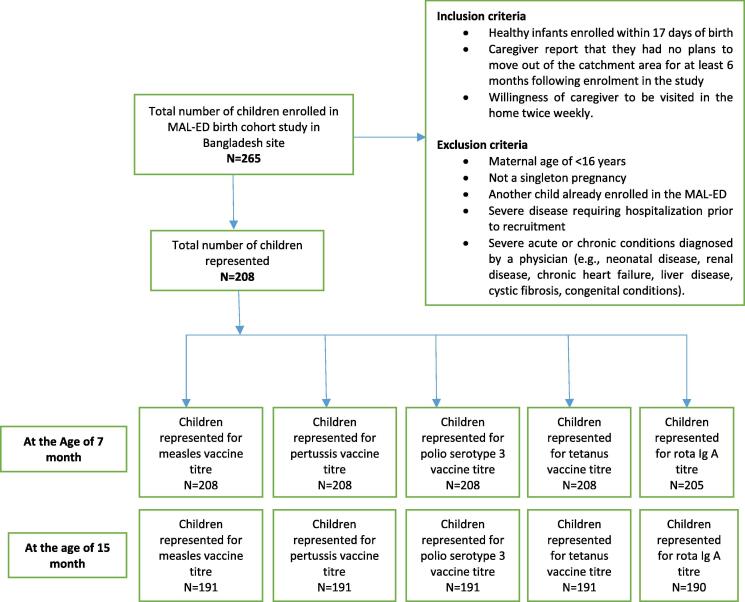
Study flow diagram.

The study was conducted in the MAL-ED (Etiology, Risk Factors, and Interactions of Enteric Infections and Malnutrition and the Consequences for Child Health) Bangladesh site birth cohort study. The study site was an underprivileged community in Bauniabadh slum, Mirpur, Dhaka, which was described elsewhere [14]. The study had a well-defined recruitment protocol [15]. Between February 2010 and February 2012, within 17 days of birth 265 healthy newborn children were enrolled by field workers upon visiting their respective households; and these children were followed until 24 months of age [16]. Here 208 participants’ data were available for analysis after total recruitment at 7 and 15 months of age (Fig. 1).

### 2.2 Data collection

#### 2.2.1 Dependent variables

##### 2.2.1.1 Assessment of vaccine responses

Scheduled blood collection was at 7 and 15 months of age (window extended from 2 days before to 12 days after the target collection date) to accommodate participants’ availability and illness by using standardized protocols [2], [17]. Enzyme-linked immunosorbent assays (ELISA) were used to quantitatively determine the immunoglobulin G (IgG) antibody levels to polio and measles virus; tetanus and pertussis toxoid; and the immunoglobulin A (Ig A) antibody level to rotavirus [2]. Both ELISAs had paired samples (at 7 and 15 months) and were placed on the same plate. At the Armed Forces Research Institute for Medical Studies (AFRIMS) in Bangkok, Thailand, assays for all vaccines other than OPV were conducted centrally; for poliovirus (types 1, 2, and 3) antibody neutralization assays, samples were evaluated at the WHO reference laboratories (Centers for Disease Control and Prevention [CDC], Atlanta, Georgia, for Bangladesh sites [2].

i. Measles, Tetanus, Pertussis, and Poliovirus IgG Assays

Quantitative anti-measles, anti-tetanus toxoid, and anti-pertussis toxin IgG ELISAs (Euroimmun, Lubeck, Germany) and anti-poliovirus IgG ELISAs (Genway, San Diego) were centrally procured for Bangladesh sites at AFRIMS; tests were performed on the instructions of the manufacturer [2].

ii. Rotavirus Assays

It administered quantitative anti-rotavirus serum IgG and IgA ELISAs centrally. In short, microplates were coated with IgG anti-rotavirus rabbit, and either cell lysate or virus preparedness was applied to alternating rows after washing. Eight 2-fold dilutions were made, beginning with 1:80 dilutions of the IgA and IgG serum levels. Four 2-fold dilutions were prepared of 1:20 dilutions of known reference IgA and IgG and unknown serum or plasma samples. After washing, the microplates were inserted with the serum standard dilutions and serum sample dilutions. After washing again, biotinylated rabbit antihuman IgA (for the IgA plates) or IgG (for the IgG plates) was added and then avidin–biotin-peroxidase complex was washed and inserted. O-phenylenediamine dihydrochloride substrate was applied to each well after the final wash, and the reaction with sulfuric acid stopped. The plates were read at 492 nm, and a 4-parameter fit of the transformed optical density values computed the titers.

iii. Poliovirus Neutralizing Antibody Assays

Randomized and blinded Bangladesh site serum samples were sent frozen to the CDC where the titers of the neutralization were determined. Approximately 100 median infective doses of tissue culture (TCID50) of Sabin poliovirus strains were applied to replicate wells at each dilution and mixtures incubated at 37°C for 1 h until 50,000 cells / mL of Vero cells are introduced. The cells were set, labeled, and examined for cytopathic effect after 3 days of further incubation at 37°C. In parallel for each poliovirus, positive controls were developed as “back titrations” to ensure that an appropriate amount of virus was added, with back titrations needed within the input virus range of 30–300 TCID50. The endpoint was described as the highest serum dilution showing a 50% or greater cytopathic effect reduction. A 1:8 antibody neutralizing titer was labeled protective.

Vaccine titers were measured using WHO-standardized micro neutralization assays [2]. Considering repeated longitudinal measurement of the outcome variable (vaccine response), seropositivity was assumed as measles titer > 250 U/mL, pertussis titer > 38 U/mL and > 26 U/mL at the age of 7 and 15 months respectively, tetanus titer > 100 U/mL, polio serotype 3 titers > 8 U/mL (as defined in the MAL-ED study), and seroconversion of rotavirus (after a previous exposure of a child to rotavirus) was defined as the Ig A or Ig G values > 20 U/mL [18].

##### 2.2.1.2 Vaccine coverage

The MAL-ED study itself did not conduct the vaccination, children were vaccinated at local health amenities and during vaccine campaigns [17]. The Expanded Programme on Immunization (EPI) vaccine schedule was followed for the Bangladesh site. Bacillus-Calmette-Guerin (BCG), hepatitis B, diphtheria-pertussis-tetanus (DPT), oral polio vaccine (OPV), Haemophilus influenza type B (Hib), and measles vaccine were administered; the rotavirus vaccine was not administered at the Bangladesh site [2], [19]. According to the Bangladesh EPI coverage evaluation survey 2014, 81.6 percent of the children across the country received all the scheduled vaccines by the age of 12 months following EPI-recommended age and the valid interval between the doses. By antigen, valid BCG coverage was 99.2%, Penta1 92.6%, Penta2 93.3% and Penta3 93.0%. The coverage for the three doses of OPV was 95.8 percent, 95.1 percent, and 92.7 percent, respectively [20]. The global EPI target of fully vaccinated rates of > 90% for all children, was reached in only two sites (Nepal and Bangladesh) of MAL-ED [19]. One of the reasons for the success of EPI in Bangladesh is the efforts that it undertook to create demand for immunization services through extensive program communication tools, where media like radio, television, newspaper, etc. were used to inform people of the benefits of immunization. In the MAL-ED study, there was no influence of socioeconomic status on vaccination coverage. Only 85% of children born at home were fully vaccinated compared to 97% children born at a health facility [19].

To record dates of vaccination, structured questionnaires were administered monthly. Confirmed dates and receipt of vaccination were assessed quarterly. The field workers also collected vaccine data using the vaccine information form for all vaccines administered that included vaccine record, clinical record, mother, or caregiver’s report [2]. MAL-ED field-workers regularly collected data on the duration and timing of vaccine administration during monthly household visits, preferably within a 2-day time frame of the monthly birth anniversary. During the monthly household visit, field-workers from MAL-ED registered the administered vaccines and administration dates on the Monthly Format A / B. Ideally the information was retrieved from the vaccine card; however, where there were no vaccine cards available, clinical records were used where possible. If no vaccine or clinical records were available, field workers asked the mother or caregiver whether vaccinations had been given since the previous monthly visit and asked about the form and date of administration of the vaccine. Besides, a quarterly review of the administered vaccines and dates of administration using the vaccine information form functioned as a confirmation tool for the data obtained on the monthly forms. The source of the vaccine data was also obtained for all vaccines administered (i.e., vaccine record, health record, the parent or caregiver report) [19].

##### 2.2.1.3 Vaccine efficacy

In the MAL-ED study, we conducted serological testing of the EPI (Expanded Programme on Immunization) vaccines. There is no question at all about the efficacy of the EPI vaccines. Serological testing is an accepted and useful method for finding a protective antibody level of vaccine-preventable diseases [21]. Here we have used the unstimulated vaccine titer and we looked for a protective titer level of a well-established vaccine for public health. For this, we stimulate with mitogens in vitro and look at the laboratory vaccine titer level of MAL-ED children.

#### 2.2.2 Explanatory variables

##### 2.2.2.1 Serum zinc and vitamin D levels

Blood samples were collected for measuring serum zinc, vitamin D, and inflammation-adjusted serum ferritin and retinol status at 7 and 15 months of age. Serum zinc, ferritin, and vitamin D and A levels were measured by using atomic absorption spectrometry and chemiluminescence immunoassay, respectively [16], [22]. Serum zinc was assessed as the measure of zinc status. Zinc concentration is a proxy marker and recommended to use for assessment of serum zinc status, especially for children in low-income countries [23]. Zinc deficiency was considered when plasma zinc concentrations were < 9.9 mmol/L [24]. Low serum vitamin D was defined when serum vitamin D concentration was < 50 ng/mL [25].

#### 2.2.3 Covariates measurement:

We selected the covariates based on the factors that have the potential to influence the vaccine responses [1], [26], [27] based on the previous literature and availability of data from our study. Outlines of the factors included in our analysis are provided below.

##### 2.2.3.1 Serum vitamin A and ferritin status

In the MAL-ED study, we assessed serum retinol concentration which is the standard indicator for vitamin A status [28]. Low serum vitamin A was defined when serum retinol concentration was < 20 μg/dL [29]. Iron deficiency was defined when the serum ferritin level was below 12 μg/L [30]. Ferritin is a measure of the iron store in the body in the absence of any concurrent infection [24].

##### 2.2.3.2 Morbidity status

At twice-weekly home visits, trained field workers collected illness information of every enrolled child daily and breastfeeding status from the mother by using a surveillance assessment form [31]. We evaluated 2 common illnesses - acute lower respiratory infection (ALRI) and diarrhea using standard definitions of illness onset and episodes. Diarrhea is defined as having three or more abnormally loose stools in 24 h or at least one loose stool with blood reported by the mother [16], [32]. Two or more diarrhea-free days marked separate diarrheal episodes [33]. Acute lower respiratory infection (ALRI) is defined as the presence of cough and/or difficulty in breathing plus age-specific high respiratory rate. This definition is commonly used to identify children in resource-poor settings who may benefit from treatment [16], [34]. The average of two respiratory rates (one minute for each measurement) was used, and different rates were considered high based on different age groups (<2 months of age: ≥60 breaths/minute; 2 to < 12 months of age: ≥50 breaths/minute; ≥12 months of age: ≥40 breaths/minute) [16]. At least 15 ALRI-free days are marked as separate ALRI episodes [16].

##### 2.2.3.3 Exclusive breastfeeding (EBF)

Depending upon the information obtained during the last 24 h recall at 12, 18, and 24 months of age current breastfeeding status was categorized as breastfed or not [35]. WHO guideline was used to define Exclusive Breast-Feeding (EBF) status [36]. The study staff asked the mother about the liquids the child had taken during the past 24 h. When the response followed the WHO definition of exclusive breastfeeding (no other food or drink, not even water, except breast milk (including milk expressed or from a wet nurse) for 6 months of life, but allows the infant to receive ORS, drops and syrups (vitamins, minerals, and medicines) [37], the child was considered as exclusively breastfed [16]

##### 2.2.3.4 WAMI score

Beginning at 6 months of age, socioeconomic data were collected every 6 months. The WAMI score (Water, sanitation, hygiene, Asset, Maternal education, and Income index, ranging from 0 to 1) is a socioeconomic status index. It includes access to improved water and sanitation, eight selected assets, maternal education, and household income as a representative of the socioeconomic status of the households [38]. A better socioeconomic status is indicated by a higher WAMI score [16].

##### 2.2.3.5 Under-nutrition

Every month, trained field workers followed standard anthropometric methodology to measure children’s weight using a frequently-standardized digital scale with 10gram precision (Seca, model-345, Hamburg, Germany) and recumbent length to the nearest 1 mm, [37]. Different anthropometric measurements: weight-for-age z-score (WAZ), length-for-age z-score (LAZ), and weight-for-length z-score (WLZ) were calculated by using the revised WHO growth standard (2006) [39]. The child with LAZ score, WAZ score, WLZ score less than two standard deviations of the age-specific medians classified as stunted, underweight, and wasted respectively [40]. Undernutrition was defined as having at least one of the above conditions.

### 2.3 Statistical analysis

We reported the child, maternal, household-level characteristics, and morbidity status by using mean and SD for continuous variables and frequency as a percentage for categorical variables. Median and interquartile range (IQR) were reported for non-normally distributed continuous variables. Wilcoxon rank-sum (Mann–Whitney) test was used to compare the vaccine titers in sera of MAL-ED Bangladesh children as a function of serum zinc and vitamin-D deficiencies in 7 and 15 months of age. The longitudinal data analysis was carried out using data collected from 7 to 15 months of age to make sure the availability of the highest number of variables at each time point and the temporality of the relationship between the outcome and explanatory variables. The relationship of each explanatory variable (low serum zinc and vitamin D) with the outcome variable (vaccine titers) was examined longitudinally using population-specific generalized estimating equations (GEE) [41]. The GEE technique is an expansion of the quasi-likelihood strategy which allows us to specify a working correlation matrix for the correlation within the subject of repeated answers gathered from the same respondents over time at distinct follow-up times. This results in more effective and unbiased regression parameters [16]. Initially, each explanatory variable (low serum zinc and vitamin D level) was used in the GEE model separately to determine its unadjusted effect on the outcome variable (vaccine titers). The covariates indicating the relationship with the outcome variable in the literature review were chosen for multivariable modeling. All covariates were used in the subsequent model to obtain an adjusted final model. Variance inflation factor (VIF) was estimated to detect multicolinearity and variables generating a VIF value > 5 were excluded from the analysis to eliminate the co-linearity. By estimating the adjusted ORs and their 95% CIs, we determined the strength of the association. During analysis, a probability of<0.05 was considered statistically significant. All data were analyzed by using STATA version 13.0 IC (College Station, Texas).

### 2.4 Ethical consideration

The research protocol was evaluated, reviewed, and approved by the Ethical Review Committee of the International Center for Diarrheal Disease Research, Bangladesh (icddr,b). Informed written consent was acquired from the parents or legal guardians of the participants enrolled in the study.

## 3 Results

### 3.1 Characteristics of the study population

A total to of 265 children (135 females and 130 males) were enrolled in the MAL-ED Bangladesh site. Nevertheless, we have complete information on 208 and 191 children at 7 and 15 months of age respectively for analysis. Table 1 describes the summary statistics of the participants. Gender was almost equally represented (female = 52.4%) at 7 months of age. Stunted, wasted, and underweight were 20.67%, 3.85%, and 14.42% respectively at 7 months of age. The prevalence of stunting, wasting and underweight increased with age from 7 to 15 months of age. About 19.7% of mothers were illiterate. Only 2% of mothers gave birth before 18 years of age. The average maternal BMI was 22.2 kg/m^2^. Up to one year of age breastfeeding was almost universal, exclusive breastfeeding taking place for an average of 106 days with almost 12% of children exclusively breastfeeding at the age of 7 months. Only 17.5% of children had lower serum ferritin levels and about half of the participants had vitamin A deficiency at 7 months of age. 24% and 28% of the participants had low serum zinc and vitamin D level. Morbidity data showed that the children suffered from 1.78 diarrheal episodes and average episodes of ALRI were 0.34 up to 7 months of age. More than 95% of children were vaccinated against tetanus, pertussis, and polio (at least 3 doses of vaccine) except measles and rotavirus vaccine, as the measles vaccine was given at the age of 9 months (according to EPI schedule) and the children of Bangladesh site were not vaccinated against rotavirus vaccine.

**Table 1:** Descriptive characteristics of the participants of MAL-ED Bangladesh Cohort at the age of 7 months.

Child, maternal, household level characteristics		n = 208 n (%)
Sex (female)		109 (52.4)
Stunted		43 (20.7)
Wasted		8 (3.9)
Underweight		30 (14.4)
Exclusive breastfeeding	Yes	25 (12.0)
Mother's age at 1st child birth (years)	<18 years	4 (1.9)
	18–30 years	176 (84.6)
	>30 years	28 (13.5)
Maternal education (years)	No education	41 (19.7)
	<5 years	53 (25.5)
	5 years or more	114 (54.8)
Maternal BMI	Mean ± SD	22.25 ± 3.49
EBF in days		106.86 ± 57.21
WAMI*		0.53 ± 0.12
**Serum biomarkers**		
Low serum Vitamin D (n = 203)		57 (28.1)
Low serum Zinc (n = 202)		49 (24.3)
Low serum ferritin (n = 200)		35(17.5)
Low serum retinol (Vitamin A)		100 (49.8)
**Morbidity status**		
Episodes of diarrhea	Mean ± SD	1.78 ± 1.63
Episodes of ALRI		0.34 ± 0.73
**Vaccination status (n = 208)**		
Tetanus (vaccinated)		206 (99.0)
Pertussis (vaccinated)		206 (99.0)
Polio (≥3 doses)		200 (96.2)

*The WAMI score (ranging from 0 to 1) is a measure of household socioeconomic status, including access to improved water, sanitation and hygiene; assets; maternal education; and income. A score of 1 means a better socioeconomic status.

Normal ranges: Zinc, ≥9.9 mmol/l; Ferritin, >12 μg/l; retinol, >20 μg/dL; vitamin D, >50 ng/mL.

Weight-for-age Z-score (WAZ), length-for-age Z-score (LAZ) and weight-for-length Z-score (WLZ) less than two standard deviations below the age specific medians classified as stunted, under weight and wasted.

ALRI, acute lower respiratory infection.

### 3.2 Association between vaccine titer and serum vitamin D and zinc level

At 7 months of age 202 children were tested, 24.3% of children had serum zinc concentration < 9.9 mmol/L, indicative of a low serum zinc status. These children did not differ from the children having normal serum zinc levels concerning measles, tetanus, and pertussis vaccine titers, except rotavirus Ig A and polio serotype 3. And at 15 months of age 189 children were tested, among them 38 children showed low serum zinc concentration who had a lower level of tetanus, pertussis, and polio 3 vaccine titers but surprisingly the children having normal serum zinc level showed lower measles and rotavirus Ig A vaccine titers, which were not statistically significant (Table 2).

**Table 2:** Vaccine titers in sera of MAL-ED Bangladesh children as a function of serum zinc and Vitamin-D deficiencies.

Nutritional parameter	Cut-off	n	Measles	Tetanus	Rota IgA	Pertussis	Polio3
	**at the age of 7 month**						
**Serum Zinc**	<9.9 mmol/L	49	41 (32, 65)	1405 (658, 1953)	16 (1, 312.5)	20 (5, 82)	407 (80, 815)
	≥9.9 mmol/L	153	39 (32, 57)	1463 (554, 2744)	6 (1, 87)	20 (5,64)	512 (102, 1024)
**p-value**			0.612	0.749	0.900	0.715	0.536
**Serum vitamin-D**	<50 nmol/L	57	37 (30, 50)	1323 (658, 2078)	31 (2, 166)	26 (7, 83)	512 (161, 1024)
	≥50 nmol/L	146	41.5 (33, 63)	1489 (554, 2679)	5 (1, 77)	19 (3, 64)	512 (80, 1024)
**p-value**			**0.027**	0.522	**0.043**	0.325	0.953
	**at the age of 15 month**						
**Serum Zinc**	<9.9 mmol/L	38	704 (296, 1683)	274 (88, 838)	176 (69, 608)	2 (0,17)	459.5 (64, 1024)
	≥9.9 mmol/L	151	577 (226, 1937)	627 (258, 1017)	127 (43, 381)	8 (0,19)	512 (80, 1024)
**p-value**			0.461	0.192	0.115	0.315	0.782
**Serum vitamin-D**	<50 nmol/L	91	668 (256, 2154)	521 (195, 952)	182 (40, 580)	7 (0, 20)	512 (64, 1024)
	≥50 nmol/L	98	589.5 (191, 1878)	633 (201, 1052)	119 (52, 248)	4.5 (0, 17)	407 (80, 1024)
**p-value**			0.179	0.477	0.178	0.703	0.784

(Median vaccine titer and IQR*).

*IQR: Interquartile range.

At 7 months of age 203 children were tested, among them 28.1% showed serum vitamin D concentration < 50 nmol/L, indicative of a low serum vitamin D status. The children having normal serum vitamin D levels had higher measles vaccine titer (p value = 0.027), but lower serum rotavirus Ig A titer (p value = 0.043). Children at 15 months of age who had normal serum vitamin D levels showed lower levels of measles, rotavirus Ig A, pertussis, and polio vaccine titer except tetanus.

**Fig. 2:**
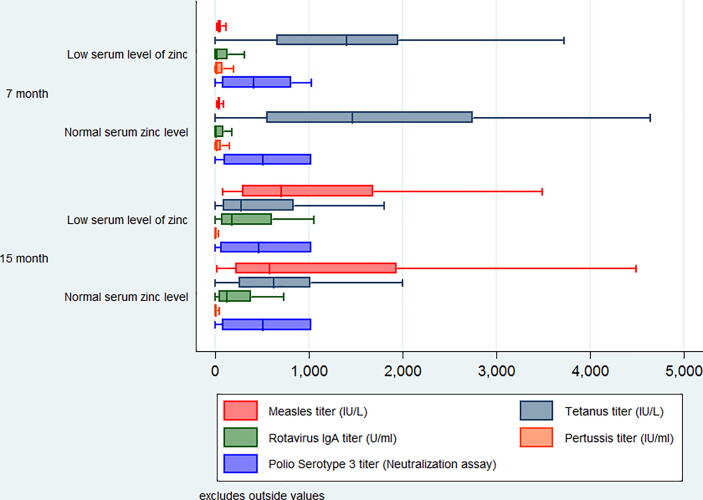
Vaccine titers in sera as a function of serum zinc status at the age of 7 and 15 months.

**Fig. 3:**
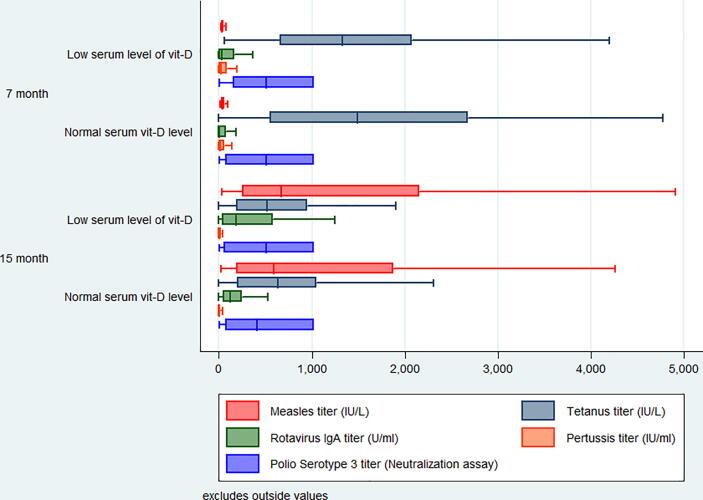
Vaccine titers in sera as a function of serum vitamin D status at the age of 7 and 15 months.

Fig. 2 represents the pattern of different vaccine titers at 7 and 15 months of age. Over the period, the trend of tetanus vaccine titer differs at both 7 and 15 months of age concerning serum zinc level. As the children were vaccinated against measles at 9 months of age (according to the EPI schedule), the measles vaccine titer was very low at 7 months of age. But rotavirus Ig A titer was higher among the low serum zinc and vitamin D level at 15 months. Polio serotype 3 vaccine titer was almost the same as low serum vitamin D level at both 7- and 15-months age groups (Fig. 3). Surprisingly the children having lower vitamin D levels showed higher measles titer at 15 months.

Table 3 presents the results of GEE modeling. The unadjusted GEE model showed a significant association between serum zinc level with positive tetanus vaccine titer and serum vitamin D level with positive rotavirus Ig A and measles vaccine titer (p < 0.05). Bivariate GEE modeling was performed longitudinally, considering all variables once at a time. In the unadjusted model, a significant positive association of serum zinc with tetanus vaccine titer and an inverse association between serum vitamin D with measles and rotavirus were found. However, after adjustment with age, gender, birth weight, WAMI score, diarrhea, ALRI, exclusive breastfeeding in days, serum ferritin, serum vitamin A, and undernutrition the multivariable model showed only a significant positive association of serum zinc level with tetanus vaccine titer [OR = 1.84; (95% CI = 1.07–3.17; p-value < 0.05)]. No association was found between the rest of the vaccine titers (for polio serotype 3, pertussis toxoid, measles virus, and the Ig A antibody of rotavirus) with serum vitamin D and zinc level (p-value for all was > 0.05).

**Table 3:** Association of vaccine titers with low serum zinc and vitamin D levels: Results of generalized estimating equation modelling (dependent variable- vaccine titers seropositive vs. seronegative).

Vaccine Titers (Seropositivity)	Unadjusted OR (95% CI)	p-value		Adjusted OR (95% CI) *	p-value
	**Serum Zinc**				
**Measles**	0.85 (0.55–1.31)	0.46		0.63 (0.29–1.37)	0.239
**Pertussis**	0.76 (0.47–1.23)	0.27		0.78 (0.47–1.28)	0.326
**Tetanus**	1.82 (1.03–3.24)	0.039		1.84 (1.07–3.17)	0.028
**Polio Serotype 3**	1.16 (0.59–2.24	0.67		1.05 (0.52–2.09)	0.896
**Rota IgA**	0.87 (0.52–1.45	0.58		0.70 (0.39–1.28)	0.250
	**Serum vitamin D**				
**Measles**	0.55 (0.38–0.79)	0.001		0.73 (0.39–1.38)	0.336
**Pertussis**	1.29 (0.83–2.01)	0.25		0.89 (0.55–1.43)	0.630
**Tetanus**	1.43 (0.84–2.44)	0.19		1.06 (0.62–1.80)	0.837
**Polio Serotype 3**	0.89 (0.49–1.65)	0.73		1.11 (0.58–2.13)	0.751
**Rota IgA**	0.49 (0.31–0.77)	0.002		0.61 (0.35–1.04)	0.068

* adjusted for Age, gender, birthweight, WAMI score, diarrhea, ALRI, exclusive breastfeeding in days, serum ferritin & retinol and under nutrition (stunting, wasting, underweight).

Seropositivity was defined as measles titer > 250 U/mL, pertussis titer > 38 U/mL and > 26 U/mL at the age of 7 & 15 months respectively, tetanus titer > 100 U/mL, polio serotype 3 titer > 8 U/mL, seroconversion of Rota IgA value was > 20 U/mL.

OR: odds ratio, CI: confidence interval.

## 4 Discussion

This MAL-ED prospective longitudinal study of children in Bangladesh's urban slum made it possible to analyze the effect of micronutrient deficiency on the response of EPI vaccines.

We found a positive association of serum zinc level with tetanus vaccine titer. The impact of zinc on the immune response to tetanus toxoid vaccines has been researched in Ecuadorian children between 0 and 59 months of age [42]. In this observational study, there was no difference between zinc deficient and zinc-sufficient children for tetanus toxoids in terms of serum antibody titers and the proportion of protecting antibodies. Overall, zinc deficiency and zinc supplementation have no measurable impact on tetanus toxoid vaccine responses. However, animal trials showed that zinc deficiency influenced the T-dependent antibody response to more than T-independent responses. But both responses were also shown to be impaired by zinc deficiency following subsequent vaccination (e.g. booster), implying that zinc status affects immunological memory [1]. One systematic review showed that there was no association with serum zinc level and BCG, Hib, pneumococcal, and diphtheria vaccine response in both children and adults [1].

Studies have shown that supplementation with vitamin D has increased the response to different vaccinations [43]. One study found enhanced tetanus toxoid specific IgG with vitamin D after vitamin D supplementation on tetanus toxoid booster vaccination [44]. But in our study, we did not find any association between serum vitamin D and tetanus vaccine response.

Very few studies have investigated the effect of vitamin D on human vaccine responses. Most of the other research was carried out in immunocompromised patients, particularly in patients with hemodialysis, as vitamin D deficiency is frequent in this group [1]. A cross-sectional analysis of NHANES, 2001–2004 among the US adult population showed a negative association between serum vitamin D levels, and measles antibody titers [45]. But in another cohort, they proposed that particular allelic differences and haplotypes of the vitamin D receptor genes could affect adaptive immune responses to measles vaccines among children in the United States [46]. The above studies and our present study findings prompted us to speculate that vitamin D may play a regulatory role in an immune response. The mechanism of how vitamin D levels influence the outcome of the response to the vaccine remains unknown to date. Activating an immune response, either by natural infection or vaccine, involves a responsive interplay between innate and adaptive immune systems [47]; Both these immune arms may be impaired by vitamin D. 25(OH)D has been shown to cause intracranial activity in antigen-stimulated dendritic cells which can then inhibit their maturation and thus prevent T-cell and B-cell proliferation in the downstream [48], [49], [50]. Besides, 1,25 (OH)D has been found to have a potent and direct effect on the B-cell response that hampers B-cell proliferation, class-switched B-cell memory generation, plasma cell differentiation, and immunoglobulin generation. 25(OH)D exhibits similar properties but at concentrations comparatively higher than 1,25(OH)D [51]. Such evidence indicates inadequate levels of vitamin D may result in a more ardent immune response. This not only coincides with previous research but also confirms the inverse relationship between the levels of vitamin D and the vaccine titers found in our study.

In our study, though rotavirus vaccination was not done, we found rotavirus IgA in the children. Children in Dhaka, Bangladesh as young as one month have been found on average to have two enteric pathogens present in stool samples even without overt diarrhea. It is possible that such subclinical infections could be causing a chronic innate immune activation localized to the gut. Among the 5 sites without the vaccine in MAL-ED, the protection conferred by prior infection was seen. The incidence of rotavirus infection during the first year of life was highest in the Bangladesh site. Prior rotavirus infections offered significant protection against subsequent infections. In MAL-ED, sites without vaccination, children with 1, 2, and 3 infections had 43%, 62%, and 74% protection from subsequent infection (p-value < 0.001) in sites not using vaccine [18].

We found scientific evidence showing the positive effect of zinc supplementation in rotavirus diarrhea [52]. A study from Bangladesh shows that zinc supplementation increases seroconversion to biocidal antibodies and therefore can enhance the effectiveness of oral cholera vaccine in children [53]. In another research of MAL-ED pooled data on rotavirus vaccine response, despite low rates of stool detection, serology showed that>90% of the study cohort had > 20 units of Ig A or IgG antibodies by 7 months of age. Almost 90% and 95% of children were exposed by serology (serological testing positive, Ig A or IgG > 20 U/mL) at the age of 7 and 15 months of age respectively in Bangladesh site (whereas Bangladesh is not using rotavirus vaccine) [18]. In the animal model, the establishment of the antiviral response by rotavirus depends on the signaling pathway of RIG-I (retinoic acid-inducible gene I), vitamin D is strongly connected to the signaling pathway of RIG-I, which mediates innate immune responses [54]. We did not find any reasons for the reduced seroconversion rates in developing countries for rotavirus vaccines. Speculation also included a non-specific function for a particular deficiency of micronutrients [55]. A study from Bangladesh showed that additional zinc increased the geometric mean titers of RotaShield immunized children [55]. So, further study should be conducted to evaluate the effect of serum vitamin D and zinc deficiency on the rotavirus vaccine response.

In terms of microbial exposure, the differentiation of responses caused by natural exposure rather than pertussis vaccination presented a further challenge in assessing the response of the pertussis vaccine [2], and probably the same happened for rotavirus vaccination, as in Bangladesh site rotavirus vaccine was not given [19].

In the event of polio vaccine response, less reliable and lower antibody responses were induced when the vaccine was administered to children in developing countries [55]. And for serotype 3 this was particularly true. The reasons for the less coherent response among children in developing countries are not evident; though most studies focused on the potential role of competing for enteric virus infections that may interfere with enteric vaccine virus infection and there might be a possibility that breast milk antibodies neutralize the vaccine virus. [55]. Some studies also assessed the function of nutritional status, such as the impact of vitamin A on the response of the polio vaccine, but not zinc or vitamin D status.

This study also reported its limitations. As it was conducted in a slum area and therefore the results may not apply to the rest of the non-slum environment. They also include failure to assess interference with serotype 2 (probably minimal because of the present trivalent OPV formulation), failure to evaluate mucosal immune responses [56] preventing transmission, and absence of maternal antibody, history of maternal infection, and gestational age of the children data from MAL-ED [1]. There was also a lack of data on sunlight exposure and dietary supplement for vitamin D and seasonal and diurnal variation of vitamin D level. Most importantly, the observational nature of the study led to limitations arising from blood collection for vaccine response was limited to only two-time points. Comparison to children from a higher socioeconomic status in Dhaka could add to the understanding of serum zinc and vitamin D status on vaccine response.

## 5 Conclusion

In summary, we address the positive association between serum zinc levels and the tetanus vaccine response. This finding implies the need to improve zinc nutrition among children in Bangladesh as well as in other countries faced with a similar burden of zinc deficiency at the population level. Zinc supplementation enhanced the immune response to tetanus vaccination. An integrated approach employing targeted supplementation, fortification, and dietary strategies must be used to maximize the likelihood of tetanus vaccination. The strategies must also be integrated with the ongoing Expanded Program on Immunization (EPI) in Bangladesh. Successful implementation and sustainability of these strategies will improve zinc nutrition among Children.

## 6 Contributors

Tahmeed Ahmed developed the MAL-ED project and was the site principal investigator. Mustafa Mahfuz developed the data collections tools and managed the field acitvities. Md. Ashraful Alam and Md. Ahshanul Haque managed the data set and provided technical support. Rina Das conceived the hypothesis, analyzed the data, developed the tables/graphs presented here, and wrote the first draft of the manuscript. Subhasish Das, Mohammod Jobayer Chisti, Dinesh Mondal and Tahmeed Ahmed critically reviewed the manuscript and provided intellectual inputs. All authors contributed to the final version of the paper.

## Funding

The MAL-ED study was funded by the University of Virginia with support from MAL-ED Network Investigators in the Foundation of National Institute of Health, Fogarty International Centre with overall support from the Bill and Melinda Gates Foundation. We acknowledge the contribution of icddr,b’s core donors including the Government of the People’s Republic of Bangladesh, Global Affairs Canada, Canada; Swedish International Development Cooperation Agency and the Department for International Development, UK Aid for their continuous support and commitment to the icddr,b’s research efforts.

## Declaration of Competing Interest

The authors declare that they have no known competing financial interests or personal relationships that could have appeared to influence the work reported in this paper.
