# Supplementary material for: Evaluating association of vaccine response to low serum zinc and vitamin D levels in children of a birth cohort study in Dhaka
Source: Vaccine. 2021 Jan 3;39(1):59–67. doi: 10.1016/j.vaccine.2020.10.048 (PMC7735373; doi:10.1016/j.vaccine.2020.10.048)
Supplement: Supplementary data 1 [file mmc1.docx]

**Supplementary table 1**. Association of low serum zinc with linear growth, wasting and stunting

|  | **Linear growth (Stunting)** | | **Underweight** | | **Wasting** | |
| --- | --- | --- | --- | --- | --- | --- |
| **Indicators** | **aOR (95%CI)** | **p-value** | **aOR (95%CI)** | **p-value** | **aOR (95%CI)** | **p-value** |
| Low serum zinc | 0.82 (0.49-1.37) | 0.450 | 0.83 (0.44-1.55) | 0.557 | 0.94 (0.37-2.43) | 0.903 |
| Age in month | 1.18 (1.10-1.25) | <0.001 | 1.18 (1.09-1.28) | <0.001 | 1.25 (1.11-1.41) | <0.001 |
| Sex (female) | 0.35 (0.19-0.66) | 0.001 | 0.73 (0.35-1.48) | 0.379 | 1.23 (0.45-3.73) | 0.640 |
| Birth weight (kg) | 0.14 (0.06-0.31) | <0.001 | 0.05 (0.02-0.14) | <0.001 | 0.09 (0.02-0.35) | 0.001 |
| WAMI score | 0.03 (0.002-0.41) | 0.008 | 0.02 (0.001-0.54) | 0.019 | 0.01 (0.00-1.20) | 0.060 |
| Diarrhoea episodes | 1.01 (0.91-1.11) | 0.889 | 1.01 (0.89-1.13) | 0.886 | 0.94 (0.81-1.11) | 0.489 |
| ALRI episodes | 0.89 (0.69-1.15) | 0.362 | 0.82 (0.59-1.13) | 0.227 | 0.93 (0.59-1.47) | 0.765 |
| Exclusive breastfeeding | 1.00 (0.99-1.01) | 0.937 | 1.00 (0.99-1.01) | 0.179 | 1.00 (0.99-1.01) | 0.728 |
| Serum Ferritin | 1.00 (0.99-1.01) | 0.054 | 1.01 (0.99-1.01) | 0.097 | 1.01 (1.00 -1.02) | 0.009 |
| Serum Retinol | 1.02 (0.99-1.05) | 0.301 | 0.98 (0.94-1.01) | 0.223 | 0.98 (0.93-1.03) | 0.455 |

aOR: adjusted Odds ratio, ***** adjusted for age, sex, birth weight, WAMI score, diarrhea, ALRI, exclusive breastfeeding in days, serum ferritin and retinol; CI: confidence interval; WAMI score (ranging from 0 to 1) is a measure of household socioeconomic status, including access to improved water, sanitation and hygiene; assets; maternal education; and income. A score of 1 means a better socioeconomic status.

**Supplementary table 2. Descriptive table on sub group analysis for children having both stunting and wasting**

|  | **At the age of 7 months** | | **At the age of 15 months** | |
| --- | --- | --- | --- | --- |
|  | **Wasted and stunted** | **others** | **Wasted and stunted** | **others** |
| **Low serum zinc** | 1 | 48 | 2 | 36 |
| **Low serum vit D** | 2 | 55 | 5 | 86 |

**Table 3. Association of vaccine titers with low serum zinc and vitamin D levels: Results of generalized estimating equation modelling (dependent variable- vaccine titers seropositive vs. seronegative)**

| **Vaccine Titers**  **(Seropositivity)** | **Unadjusted OR (95% CI)** | **p-value** | |  | **Adjusted OR (95% CI) *** | **p-value** |
| --- | --- | --- | --- | --- | --- | --- |
|  |  | | **Serum Zinc** | | | |
| Measles | 0.85 (0.55-1.31) | 0.46 | |  | 0.63 (0.29-1.37) | 0.239 |
| Pertussis | 0.76 (0.47-1.23) | 0.27 | |  | 0.78 (0.47-1.28) | 0.326 |
| Tetanus | 1.82 (1.03-3.24) | 0.039 | |  | 1.84 (1.07-3.17) | 0.028 |
| Polio Serotype 3 | 1.16 (0.59-2.24 | 0.67 | |  | 1.05 (0.52-2.09) | 0.896 |
| Rota IgA | 0.87 (0.52-1.45 | 0.58 | |  | 0.70 (0.39-1.28) | 0.250 |
|  |  | | **Serum vitamin D** | | | |
| Measles | 0.55 (0.38-0.79) | 0.001 | |  | 0.73 (0.39-1.38) | 0.336 |
| Pertussis | 1.29 (0.83-2.01) | 0.25 | |  | 0.89 (0.55-1.43) | 0.630 |
| Tetanus | 1.43 (0.84-2.44) | 0.19 | |  | 1.06 (0.62-1.80) | 0.837 |
| Polio Serotype 3 | 0.89 (0.49-1.65) | 0.73 | |  | 1.11 (0.58-2.13) | 0.751 |
| Rota IgA | 0.49 (0.31-0.77) | 0.002 | |  | 0.61 (0.35-1.04) | 0.068 |

***** adjusted for Age, gender, birthweight, WAMI score, diarrhea, ALRI, exclusive breastfeeding in days, serum ferritin & retinol and under nutrition (stunting, wasting, underweight)

Seropositivity was defined as meseals titer > 250 U/mL, pertussis titer > 38 U/mL and > 26 U/mL at the age of 7 & 15 months respectively, tetanus titer > 100 U/mL, polio serotype 3 titer >8 U/mL, seroconversion of Rota IgA value was > 20 U/mL

OR: odds ratio, CI: confidence interval
